# Using an implementation science approach to implement and evaluate patient-reported outcome measures (PROM) initiatives in routine care settings

**DOI:** 10.1007/s11136-020-02564-9

**Published:** 2020-07-10

**Authors:** Angela M. Stover, Lotte Haverman, Hedy A. van Oers, Joanne Greenhalgh, Caroline M. Potter, On behalf of the ISOQOL PROMs/PREMs in Clinical Practice Implementation Science Work Group, Sara Ahmed, Joanne Greenhalgh, Elizabeth Gibbons, Lotte Haverman, Kimberly Manalili, Caroline Potter, Natasha Roberts, Maria Santana, Angela M. Stover, Hedy van Oers

**Affiliations:** 1 Department of Health Policy and Management, University of North Carolina at Chapel Hill, 1103-D McGavran-Greenberg Hall, CB # 7411, Chapel Hill, NC 27599 USA; 2 Lineberger Comprehensive Cancer Center, University of North Carolina at Chapel Hill, Chapel Hill, USA; 3 Psychosocial Department, Emma Children’s Hospital, Amsterdam UMC, University of Amsterdam, Amsterdam, The Netherlands; 4 School of Sociology and Social Policy, University of Leeds, Leeds, UK; 5 Nuffield Department of Population Health, University of Oxford, Oxford, UK

**Keywords:** Patient-reported outcome measures, Quality of life, Implementation science, Clinical practice, Routine care

## Abstract

**Purpose:**

Patient-reported outcome and experience measures (PROMs/PREMs) are well established in research for many health conditions, but barriers persist for implementing them in routine care. Implementation science (IS) offers a potential way forward, but its application has been limited for PROMs/PREMs.

**Methods:**

We compare similarities and differences for widely used IS frameworks and their applicability for implementing PROMs/PREMs through case studies. Three case studies implemented PROMs: (1) pain clinics in Canada; (2) oncology clinics in Australia; and (3) pediatric/adult clinics for chronic conditions in the Netherlands. The fourth case study is planning PREMs implementation in Canadian primary care clinics. We compare case studies on barriers, enablers, implementation strategies, and evaluation.

**Results:**

Case studies used IS frameworks to systematize barriers, to develop implementation strategies for clinics, and to evaluate implementation effectiveness. Across case studies, consistent PROM/PREM implementation barriers were technology, uncertainty about how or why to use PROMs/PREMs, and competing demands from established clinical workflows. Enabling factors in clinics were context specific. Implementation support strategies changed during pre-implementation, implementation, and post-implementation stages. Evaluation approaches were inconsistent across case studies, and thus, we present example evaluation metrics specific to PROMs/PREMs.

**Conclusion:**

Multilevel IS frameworks are necessary for PROM/PREM implementation given the complexity. In cross-study comparisons, barriers to PROM/PREM implementation were consistent across patient populations and care settings, but enablers were context specific, suggesting the need for tailored implementation strategies based on clinic resources. Theoretically guided studies are needed to clarify how, why, and in what circumstances IS principles lead to successful PROM/PREM integration and sustainability.

**Electronic supplementary material:**

The online version of this article (10.1007/s11136-020-02564-9) contains supplementary material, which is available to users.

## Introduction

Patient-reported outcome and experience measures (PROMs/PREMs) are well established in research for many health conditions [1, 2], but barriers persist for implementing them in routine care. PROMs are reports of how patients feel and function that come directly from individuals with a health condition [3], while PREMs assess patient experiences of treatment (e.g., satisfaction with care) [4]. When used during care delivery, PROMs improve communication between clinicians and patients about symptoms and quality of life [5, 6], which may improve service use and survival [1, 2, 7–11]. Reviewing PROMs with patients during clinical visits can also increase satisfaction with care scores [12]. PREMs are commonly used as performance metrics to evaluate the quality of care delivered [13], but PROM-based performance metrics are a growing trend [14]. Before clinical benefits can be realized, however, PROMs/PREMs need to be implemented into care delivery.

There is wide variation in how PROMs/PREMs are implemented [15, 16] and in the resulting impact on processes and outcomes of care [1, 2, 5, 6]. Prior research has documented the limited uptake of PROMs/PREMs and barriers to their implementation in routine care settings [17–21]. Implementation science (IS) offers a potential way forward, but its application has been limited for PROMs/PREMs. IS is the systematic study of methods to integrate evidence-based practices and interventions into care settings [22, 23]. IS aims to make the process of implementation more systematic, resulting in a higher likelihood that health innovations like PROMs/PREMs are adopted in clinics.

Part of IS’s appeal are the theories and frameworks guiding the translation process from research to practice [24–26], but there are dozens focused on health care [26]. IS frameworks and theories draw on diverse academic disciplines including psychology, sociology, and organizational science, and therefore differ in assumptions about the primary drivers of implementation processes and potential explanatory power. Frameworks identify and categorize key barriers and enablers, while theories tend to have more explanatory power because they specify practical steps in translating research evidence into practice. IS is still an emerging field, and descriptive frameworks are the most common, as outlined in Nilsen’s typological classification of theoretical approaches [24].

**Fig. 1:**
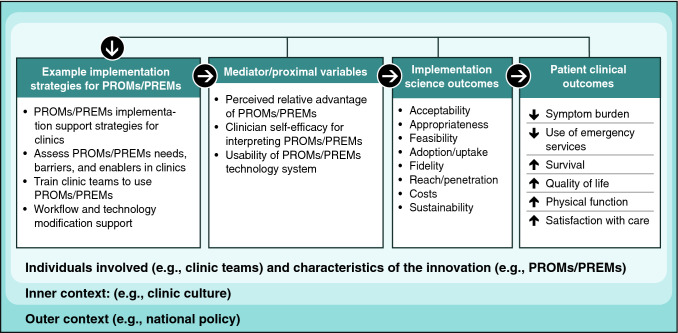
Relationships between PROM/PREM implementation strategies, implementation science outcomes, and patient outcomes

In addition to explanatory features, IS frameworks and theories may also provide a menu of potential solutions to barriers called “implementation strategies.” These strategies are actions purposively developed to overcome barriers that can be tailored to the local context [27, 28]. Figure 1 shows how implementation strategies are used to influence proximal mediators such as clinician self-efficacy for using PROM/PREMs, which impact IS outcomes for PROM/PREM implementation (e.g., Proctor’s outcomes [29]), and in turn improve clinical and health services outcomes.

Our paper is organized into three sections. First, we describe and compare IS frameworks used in four case studies. We then summarize cross-study findings on the barriers, enablers, implementation strategies, and evaluation approaches. We also derive example metrics specific to evaluating PROM/PREM implementation initiatives to support standardization. Finally, we consider the implications of these findings for future research and practice.

**Fig. 2:**
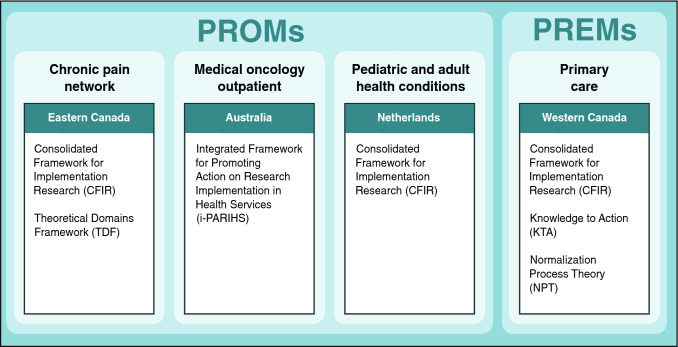
Four case studies

We compare four case studies of PROMs/PREMs implementation (see Fig. 2) that draw on established IS frameworks or theories (each case study has a stand-alone paper in this issue: [30–33]). Three case studies implemented PROMs for monitoring symptoms, psychosocial functioning, and/or health-related quality of life: (1) pain clinics in Canada [30]; (2) oncology clinics in Australia [31]; and (3) pediatric/adult clinics for chronic conditions in the Netherlands [32]. The fourth case study is planning PREMs implementation in primary care clinics in Canada [33].

## Theoretical approaches used in case studies

Across case studies, five well-established IS frameworks or theories widely used in health care settings were applied (Table 1).

**Table 1:** Key features of widely used implementation science frameworks or theories

Implementation framework or theory	Nilsen [24] classification	Constructs influencing implementation	Case stud(ies)
Consolidated Framework for Implementation Research (CFIR) www.cfirguide.org [34, 35]	Determinant framework: categorizes implementation barriers/enablers	*Characteristics of intervention or practice* (e.g., evidence, complexity, cost) *Outer setting* (e.g., patient needs, policies) *Inner setting* (e.g., organization/clinic characteristics, culture, implementation climate) *Characteristics of individuals* (e.g., clinician knowledge, self-efficacy) *Implementation process* (e.g., engaging, evaluating)	Ahmed et al. [30]: implementing ePROMs in a pain network van Oers et al. [32]: implementing ePROMs in multiple pediatric and adult health clinics Manalili and Santana [33]: implementing ePREMs for quality improvement in primary care
Theoretical Domains Framework (TDF) [36–38]	Determinant framework: categorizes implementation barriers/enablers	*Factors Influencing Clinician Behavior Change,* e.g.: Knowledge, skills Professional role/identity Beliefs about capabilities Beliefs about consequences Reinforcement Intentions/goals Environmental context and resources Social influence Memory, attention, decision influences Behavioral regulation	Ahmed et al. [30]: implementing ePROMs in a chronic pain network
Integrated framework for Promoting Action on Research Implementation in Health Services (i-PARIHS) [39–42]	Determinant framework: categorizes implementation barriers/enablers	*Successful implementation formula = Fac*^*n*^*(I + R + C)* *Fac* = *facilitation* Person or organization assigned to do work of facilitation (implementation support) *I* = *innovation* Characteristics of innovation Degree of fit with existing practice and values Usability Relative advantage Trialability/observable results *R* = *recipients* Clinical experiences/perceptions Patient experiences, needs, preferences *C* = *context* Leadership support Culture, receptivity to change Evaluation capabilities	Roberts et al. [31]: implementing paper and electronic PROMs in a medical oncology outpatient department
Knowledge to Action (KTA) www.kt.canada.org [43, 44]	Process model: describes practical steps in translating research to practice	*Knowledge creation phases:* Knowledge inquiry Knowledge synthesis Create knowledge tools *Action phases:* Determine the know/do gap Adapt knowledge to local context Assess barriers/facilitators to use Select, tailor, implement Monitor knowledge use Evaluate outcomes Sustain knowledge use	Manalili and Santana [33]: implementing ePREMs for quality improvement in primary care
Normalization Process Theory (NPT) www.normalizationprocess.org [45, 46]	Implementation theory: specifies causal mechanisms	*Coherence/sense-making* (what is the work?) *Cognitive participation* (who does the work?) *Collective action* (how do people work together to get the work done?) *Reflexive monitoring* (how are the effects of the work understood?)	Manalili and Santana [33]: implementing ePREMs for quality improvement in primary care

*PROM* paper or electronic patient-reported outcome measure, *ePROM* electronic patient-reported outcome measure, and *ePREM* electronic patient-reported experience measure

Three case studies [30, 32, 33] used the Consolidated Framework for Implementation Research (CFIR) [34, 35]. CFIR was developed from constructs identified in earlier frameworks and is widely used [35]. Its 39 constructs are organized into five multilevel domains: the intervention itself (e.g., PROMs/PREMs), outer setting (e.g., national policy context), inner setting (e.g., implementation climate in clinics), characteristics of individuals involved (e.g., clinic teams), and the implementation process. CFIR has a tool available to match barriers to implementation strategies (available at www.cfirguide.org), which was used prospectively for planning PREMs implementation [33] and retrospectively for assessing implementation of a PROMs portal for chronic conditions [32].

The case study in an integrated chronic pain care network [30] combined CFIR with the Theoretical Domains Framework (TDF) [36–38] to identify barriers and enablers for implementing PROMs. TDF is grounded in a psychology perspective on behavior change at the clinician level. Fourteen domains describe constructs such as clinician knowledge, skills, and perceptions.

The case study in oncology clinics [31] used the integrated Promoting Action Research in Health Services (i-PARIHS) framework [39–42]. i-PARIHS’ key construct of facilitation^1^ is the “active ingredient” driving successful implementation, for example, an implementation support person working with clinics to identify and overcome barriers. In its most recent development [40], i-PARIHS’ three multilevel predictors include: (1) the innovation (ways stakeholders perceive and adapt innovations like PROMs/PREMs to align with local priorities); (2) recipients (how individuals and communities of practice influence uptake of new knowledge in clinics); and (3) context (inner context such as clinic culture and outer context such as the wider health and policy system). The i-PARIHS facilitator’s toolkit [42] offers pragmatic guidance for supporting implementation in clinics.

The case study implementing PREMs in primary care clinics [43] combined CFIR with the Knowledge to Action (KTA) model [43, 44] and Normalization Process Theory (NPT) [45, 46]. KTA is a process model describing practical steps or stages in translating research into practice, with core concepts of knowledge creation and action. The action cycle is most applicable to PROM/PREM implementation, with steps for identifying the problem and selecting and enacting appropriate knowledge for addressing the problem.

Normalization Process Theory (NPT) [45, 46], developed from sociological theory, describes mechanisms of how an innovation becomes routinized. NPT’s focus is on what people actually do, rather than their intentions, by focusing on how the “work” of an innovation (such as using PROMs/PREMs in clinical practice) becomes normalized. NPT outlines four mechanisms driving implementation processes: coherence/sense-making (What is the work?), cognitive participation (Who does the work?), collective action (How do people work together to get the work done?), and reflexive monitoring (How are the effects of the work understood?).

Table 1 shows that there is some overlap of core constructs across implementation frameworks and theories and some unique features. Frameworks and theories typically emphasize particular constructs. For example, the i-PARIHS framework highlights the role of a facilitator (implementation support person) as a core construct. The oncology case study [31] that used i-PARIHS was the only one to employ a dedicated facilitator across the full implementation cycle; although the three other case studies did use implementation support teams for shorter durations. The case study in pain clinics [30] had a priori identified clinician engagement with PROMs as a key issue and chose TDF as their framework because it describes barriers specifically at the clinician level.

The case study at the pre-implementation stage for PREMs in primary care [33] drew on KTA and NPT, which both emphasize steps in the implementation process instead of describing barriers. As we later suggest, implementation theory that hypothesizes mechanisms of change (such as NPT) may be a useful guide for developing overarching strategies across stages of implementation. An overarching theoretical approach could then be supplemented with consideration of context-specific barriers and enablers for PROMs/PREMs through multilevel frameworks such as CFIR or i-PARIHS.

## Barriers and enablers in case studies

Frameworks described in the prior section are based on a foundation of barriers and enablers. The distinction between whether a concept is labeled as a barrier or enabler is a judgment based on the framework or theory being used and stakeholder perceptions [22, 28]. The label of barrier or enabler shapes the implementation approach considerably. For example, if lack of PROM/PREM technology is labeled as a barrier, an implementation strategy such as developing software with stakeholder input will be used. If existing technology is labeled as an enabler, the implementation team can tailor training and examples to the specific software.

Table 2 shows that the four case studies consistently described implementation barriers of technology, stakeholder uncertainty about how or why to use PROMs/PREMs, stakeholder concerns about negative impacts of PROM/PREM use, and competing demands from established clinical workflows.

**Table 2:** Barriers, enablers, and implementation strategies used in case studies

Country	Clinical setting	Implemented PROMs or PREMs	IS framework or theory	Implementation barriers identified	Implementation enablers identified	Implementation strategies employed
Eastern Canada [30]	Chronic pain network including primary care, rehabilitation care, and hospital-based care	ePROMs	*Primary care* Theoretical domains framework (TDF) [36–38] *Tertiary care* Consolidated framework for implementation research (CFIR) [34, 35]	*Barriers:* Primary care: • Well-defined clinical process: barriers at clinician level • Lack knowledge on how to interpret pain PROMs Tertiary care: • Variability in care process: multilevel barriers • Confidentiality concerns • Technology comfort • Perceived increase in workload and time to review PROMs • Perception PROMs may decrease patients’ satisfaction with care • PROMs not integrated in electronic health record • Cost and time to implement	*Enablers* • Existing PROM system easy for clinicians to use and accessible on all forms of devices • Rapid access to PROM results • Selected PROMs that are easy to complete and interpret • Top-down decision from clinic leadership to implement • Created business plan with health system and moved money to clinic budgets • Opinion leader support	*Strategies* Pre-implementation: • Identify barriers with clinic • Map observed barriers to evidence-based strategies Implementation: • Training workshop with clinic team (half day) • Local opinion leader with PROM knowledge provided coaching • Educational materials • Onsite tech support • Workflow redesign support • Support to help patients complete PROMs Post-implementation: • Examine potential cost savings by triaging patients more efficiently
Australia [31]	Medical oncology outpatient department	Paper and electronic PROMs	Integrated Framework for Promoting Action on Research Implementation in Health Services (i-PARIHS) [39–42]	*Barriers* • Gaps in infrastructure • Varying workflows • Clinics needed more time than anticipated to implement • Staff felt pressured with competing priorities • Past negative experiences with innovations	*Enablers* • Dedicated facilitator (implementation support role) • Rapid access to PROM results • Research funding • Peer champions for PROMs emerged naturally	*Strategies* Pre-implementation: • Stakeholder engagement about barriers and context assessments • Workflow assessment and redesign assistance Implementation: • Training/information resources • Technical support • Rapid cycle testing Post-implementation: • Audit and feedback to clinics
Netherlands [32]	Multiple pediatric and adult health conditions	ePROMs	Consolidated Framework for Implementation Research (CFIR) [34, 35]	*Barriers* • Some clinics undergoing too many change initiatives • PROMs not integrated in EHR • Stakeholders did not see relative advantage of PROMs • Compatibility • No organizational incentives	*Enablers* • Clinicians perceived value • Strong evidence PROMs improve clinical outcomes • Existing online portal is user friendly for patients and clinicians • Existing automated PROM reminders • Existing automatic and direct access to PROM results and visualization for clinicians • Existing ability for multidisciplinary clinic team members to customize PROMs based on patient age, health conditions, etc • Existing clinician self-efficacy	*Strategies* Pre-implementation: • Stakeholder engagement • PROM integration in EHR • Provided PROM recommendations based on patients’ age and condition Implementation • Training • Implementation support team available to all clinics Post-implementation: • Annual evaluation meeting with clinics • Reflecting and evaluating on what worked and did not work
Western Canada [33]	Primary care: implementing ePREMs for quality improvement	ePREMs	Knowledge to Action (KTA) [43, 44] CFIR [34, 35] Normalization Process Theory (NPT) [45, 46]	*Barriers* • Unclear stakeholder preferences and barriers • Unclear what optimal implementation strategies will be for PREMs and whether they differ from PROM strategies	*Enablers* • Research grant support • Collaboration with quality improvement specialists • National policy change: Primary care patient’s medical home encourages patient-centered communication and patient surveys to evaluate effectiveness of practice’s services	*Strategies* Pre-implementation • Stakeholder engagement to identify barriers (interviews with clinic teams) • Categorize barriers with theory and map to evidence-based implementation strategies Implementation: • Training clinic teams • Stakeholder engagement • Onsite coaching • Plan-Do-Study-Act rapid testing cycles Post-implementation: • Audit and feedback to clinics • Process evaluation

For technology, PROMs/PREMs not being integrated within electronic health record systems was repeatedly identified as a major barrier. Additional technology barriers included PROM collection systems that were difficult to use or access, and third-party systems requiring a separate login.

Stakeholder knowledge and perceptions were also identified as consistent barriers. Clinicians noted a knowledge barrier of being unsure how to interpret PROM responses and discuss them with their patients. There were also concerns that PROM use would lead to increases in workload and visit length, and the potential to decrease satisfaction with care. Roberts’ oncology clinic case study [31] also encountered clinics with prior negative experiences with innovations.

Clinical workflow barriers included entrenched workflow, competing priorities during limited clinic time, and unique workflow in every clinic, suggesting implementation strategies that work in some clinics or settings may not work in others. Less common barriers included resource gaps for treating and referring patients for symptoms detected by PROMs (oncology clinic case study) and confidentiality concerns for PROMs/PREMs (pain clinic case study).

**Fig. 3:**
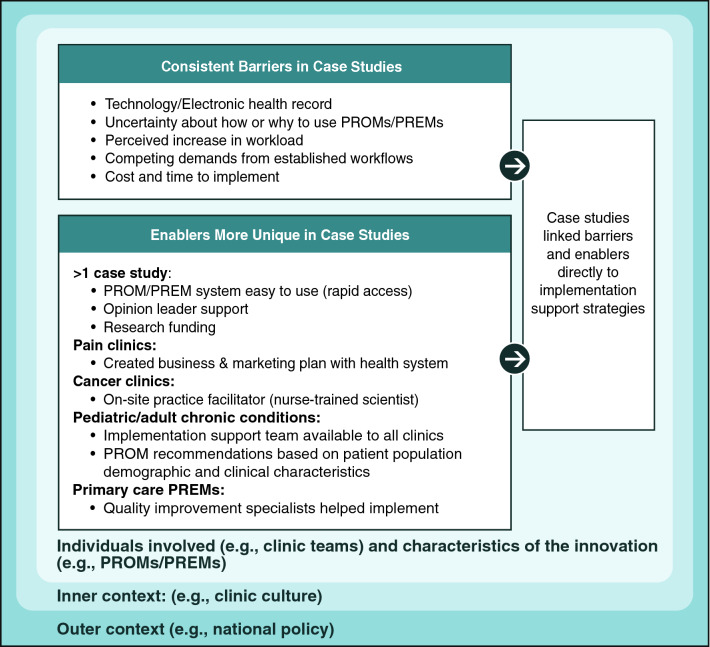
PROM/PREM barriers and enablers in case studies

Figure 3 shows that enablers varied more across case studies than barriers, suggesting that solutions were being tailored to each clinic and its resources (inner context). Common enabling factors included designing PROM/PREM technology systems to be easy for clinicians to use and enabling automatic access to PROM results for use at point-of-care. More unique enablers capitalized on local resources, such as peer champions, availability of a nurse scientist to provide long-term implementation support in an oncology clinic, and a research team working with a health system to create a pain PROM business plan and move resources (including money) to clinics. Two case studies were enabled with research grant support.

## Implementation strategies in case studies

**Fig. 4:**
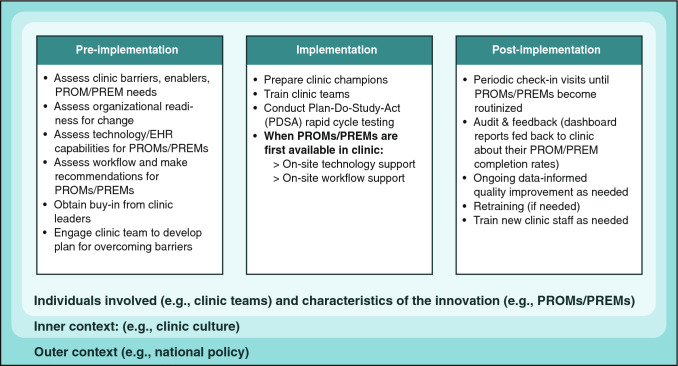
Implementation strategies used in case studies, shown by implementation stage

Case study authors matched PROM/PREM barriers and enablers encountered in clinics directly to specific implementation strategies. Figure 4 shows that implementation strategies changed during pre-implementation, implementation, and post-implementation stages and were influenced by contextual factors. During pre-implementation, case studies engaged stakeholders and clinic leaders, and assessed barriers, enablers, PROM/PREM needs, and workflow. They also engaged clinic teams to develop tailored implementation strategies. During the implementation stage, all case studies trained clinic teams on using and interpreting PROMs/PREMs and provided onsite assistance for technology and workflow. Support ranged from low intensity with one training session and a few support visits to high-intensity facilitation conducted onsite and long term (> 6 months). The pain clinic case study [30] also developed strategies to increase clinic teams’ perceptions of acceptability of PROMs through a media campaign for clinic teams. Post-implementation, all case studies continued contact with clinics, typically through visits. Three case studies also used audit and feedback where dashboard reports were fed back to clinics about their PROM/PREM completion rates. If completion rates were low, additional support was provided to clinics to identify and overcome new or ongoing barriers, suggesting that post-implementation support may be key to sustaining PROM/PREMs in clinics.

## Evaluating PROM/PREM implementation initiatives

Three case studies [30–32] used aspects of Proctor’s IS outcomes [29] to evaluate PROMs and one case study [33] used the RE-AIM framework [47, 48] to evaluate PREMs, but the degree of application and operationalization were inconsistent. Table 3 shows that Proctor’s IS framework and RE-AIM have overlapping concepts for reach/penetration, adoption, and sustainability/ maintenance. Unique to Proctor’s list are acceptability, appropriateness, feasibility, fidelity, and cost [29]. Unique to RE-AIM are effectiveness and “implementation” [47, 48].

**Table 3:** Comparison of implementation science frameworks used for evaluation

IS evaluation framework	Construct to evaluate	Construct definition	Similar construct	Case studies
Proctor’s outcomes [29]	Acceptability	Extent to which implementation stakeholders perceive innovation to be agreeable or palatable	Satisfaction	Ahmed et al. [30]: implementing PROMs in a chronic pain network Roberts et al. [31]: implementing PROMs in routine cancer care van Oers et al. [32]: implementing PROMs for pediatric and adult clinics treating chronic conditions
	Appropriateness	Perceived fit, relevance, or compatibility of innovation for given practice setting	Compatibility, usefulness	
	Adoption	Intention, initial decision, or action to employ innovation by service settings (proportion and representativeness)	Uptake	
	Feasibility	Extent to which innovation can be successfully used or carried out within given setting	Practicability	
	Reach/penetration	Extent to which target population is reached	Service penetration	
	Fidelity	Degree to which innovation or implementation strategy delivered as intended	Adherence	
	Costs	Financial impact of innovation, including costs, personnel, and clinic and patient time necessary for treatment delivery, or cost of implementation strategy	Cost–benefit, cost-effectiveness	
	Sustainability	Extent to which innovation is maintained as intended and/or institutionalized within service setting’s ongoing operations	Maintenance, institutionalized	
Reach, effectiveness, adoption, implementation, and maintenance (RE-AIM) www.re-aim.org [47, 48]	Reach	Extent to which target population is reached	Penetration	Manalili and Santana [33]: implementing PREMs for quality improvement in primary care
	Effectiveness	Impact of innovation on important outcomes, including potential negative effects, quality of life, and economic		
	Adoption	Absolute number, proportion, and representativeness of settings and intervention agents (people who deliver the program) who are willing to initiate a program	Uptake	
	Implementation	• At setting level: intervention agents’ fidelity to various elements of innovation’s protocol, including consistency of delivery as intended and time and cost of intervention • At individual level: use of intervention strategies		
	Maintenance	• At setting level: extent to which an innovation becomes institutionalized/part of routine practices and policies • At individual level: Long-term effects of innovation on outcomes 6+ months after most recent contact	Sustainability, institutionalized	

Case studies used a range of 2–6 evaluation constructs, typically obtained with qualitative methods. Given the use of Proctor’s outcome framework [29] in three out of four case studies, it may be a viable method to standardize PROM/PREM evaluation. Its constructs of acceptability and appropriateness of PROMs/PREMs for a specific clinic were the most common evaluation outcomes, and were assessed with stakeholder interviews. The remaining six Proctor outcomes were used less frequently, in part due to their applicability in later stages of implementation.

To support standardizing evaluation metrics, we derived Table 4 to describe how Proctor’s IS outcomes [29] can be evaluated specifically for PROM/PREM implementation initiatives. Table 4 makes an important distinction that evaluation metrics are different for perceptions of the *innovation* (PROMs/PREMs) vs. *implementation effectiveness*. For example, *innovation* feasibility for PROMs/PREMs may involve a pilot study of tablet vs. paper administration, but an example metric for *implementation* feasibility is the percentage of clinics completing PROM/PREM training.

**Table 4:** Implementation science metrics for evaluating PROM implementation initiatives in routine care settings

Implementation science construct	Evaluating*** perception of the innovation*** (PROMs)	Evaluating the ***implementation strategies***
Acceptability	*Patients and clinicians* • % willing to recommend PROMs to other patients • % reporting PROMs helpful in discussing symptoms/symptom management • % reporting ease of use and comprehensibility for PROMs and technology systems	• Stakeholder perceptions of acceptability of implementation strategies (e.g., PROM training session is appropriate length) • Barriers and enablers for implementing PROMs • Related contextual factor: organizational readiness for change
Appropriateness	• PROM fit with patient population (e.g., literacy level, technology comfort, language(s), font size, culturally appropriate, meaningful for clinical condition) • PROM fit for clinic team (e.g., PROM easy to interpret, meaningful for clinical care, integrated in electronic health record system, linked clinical decision support) • PROM fit with clinic culture and values • Perceived relative advantage of PROMs vs. usual care • Leadership support for PROMs	• Stakeholder perceptions of clinic needs and resources for implementing PROMs • Fit of potential implementation strategies for specific clinics, their needs and resources, clinic team members, and patient population • Leadership support for implementation strategies (e.g., providing space and time for clinic team to receive training)
Feasibility	• Extent to which technology or electronic health record can be developed or modified to administer PROMs and visualize results in a meaningful way for clinicians • If collecting PROMs from home, feasibility testing considers underserved patient groups’ needs and access to internet and habits (or alternative data collection methods like interactive voice response offered) • Consent rate > 70% (if applicable) • How many and which items are missed or skipped (and identifiable patterns) • Length of time for patients to complete the PROM, comprehensibility • Rates of technical issues • Dropout rate for patients • PROM characteristics (e.g., literacy demand, number of items, preliminary psychometric properties if used in new population, validity and reliability evidence for population)	• “Action, actor, context, target, time (AACTT)” framework [62]: describe who needs to do what differently, and select fit-for-purpose strategies • % clinics completing at least one implementation activity or phase (and/or all activities and implementation phases) • Rates of technical issues for clinics • Stakeholder perceptions of which implementation strategies are possible • Stakeholder perceptions of what to include in PROM training session • Pilot study or rapid cycle testing to determine if implementation strategy is possible (e.g. whether specific workflow change possible in a clinic) • Which implementation activities were completed vs. skipped
Adoption	• % of clinics advancing to administering PROMs routinely • Representativeness of clinics willing to initiate PROMs • Underserved patient groups (e.g., older patients) complete PROMs at similar rates to clinic average	• Dropout rate for clinics • Representativeness of clinics completing implementation activities • Stakeholder perceptions and observations on which implementation support strategies were/were not effective in a clinic, and why • How and why clinics operationalized implementation strategies • Minor changes made to implementation strategies to fit local conditions or context (if major changes, see fidelity below) • StaRI reporting guidelines for implementation strategies [61]
Reach/penetration	• % of patient panel completing ≥ 1 PROM during defined time interval (denominator chosen appropriately: all patients with an in-person visit during time interval, etc.) • % of missing data during defined time interval (with appropriate denominator) • Informed missingness (correlated with patient demographics) • Average # PROMs completed per patient during interval	• % of clinic team participating in implementation strategies • % of clinic team attending training • % of clinic team reporting training helped them understand new role and how to implement in their workflow • Clinicians: % reporting self-efficacy for using PROMs after training
Fidelity	• Consistency of PROMs completed by patients (e.g., 80% PROM completion rate for clinic) • % of clinicians who review PROMs with patients during visits • How and why clinics adapted the innovation (e.g., changed PROM timeframe for items) • FRAME framework for reporting adaptions to interventions [49]	• FIDELITY framework [50]: report on five implementation fidelity domains (study design, training, delivery, receipt, and enactment) • How and why clinics or support personnel adapted implementation strategies (e.g., changed the PROM training format or content) • % of clinics completing all implementation activities
Cost	• Financial, personnel, and time costs to administer and review PROMs on routine basis • Technology costs	• Financial, personnel, technology, and time costs to implement PROMs • Cost of Implementing New Strategies (COINS) [64]
Sustainability	• Extent to which PROMs become normalized and routinized in a clinic’s workflow • Stakeholder perceptions • Periodically assess whether updates to PROMs are needed	• Routine data-informed feedback to clinic on PROM completion rates, missing data, and informed missingness • Provide additional implementation support to identify and overcome new or ongoing barriers (if needed) • Retraining or “booster” training or train new staff (if needed)

Bold and italic font show the important distinction between evaluating perceptions of the innovation (PROMs/PREMs) vs. evaluating implementation strategies

*ePROM* electronic patient-reported outcome measure, *AACTT* action, actor, context, target, time framework, *StaRi* standards for reporting implementation studies guidelines, *FRAME* framework for reporting adaptations and modifications-enhanced, *COINS* Cost of Implementing New Strategies (COINS) scale

Table 4 shows that the constructs of adoption, reach/penetration, fidelity, and sustainability can be measured in terms of engagement rates and milestone achievements at the clinic level. For example, *innovation* fidelity can be assessed as the percentage of clinicians who review PROMs/PREMs with patients as intended. The FRAME framework [49] can be used for reporting adaptions to interventions/innovations. However, *implementation* fidelity can be assessed as the extent to which recommended implementation strategies were adhered to and how and why clinics adapted implementation strategies. The Fidelity Framework developed by the National Institutes of Health’s Behavioral Change Consortium [50] recommends reporting on five implementation fidelity domains (study design, training, delivery, receipt, and enactment). Assessment of *innovation* costs may include personnel and clinic and patient time necessary for completing and reviewing PROMs/PREMs that can be assessed via observation or economic evaluation methods [51]. *Implementation strategy* costs can be assessed through tools such as the “Cost of Implementing New Strategies” (COINS) [52].

As Table 4 illustrates, collecting evaluation data requires careful planning and resource allocation at the start of PROMs/PREMs implementation efforts, but evaluation data are critical for gauging success, ongoing monitoring, and making improvements. Figures 1, 2, 3 and 4 also show that the implementation process and evaluation metrics are influenced by contextual factors (inner and outer context, individual involved, and characteristics of the innovation), which can be assessed to help explain evaluation results. Reviews of IS scales [53, 54] include questionnaires assessing contextual factors, but they may lack psychometric and validity evidence. An alternative is to assess contextual factors with stakeholder interviews.

## Discussion

This paper makes several important contributions to the literature. Our comparison of four case studies enabled us to identify commonalities and differences in barriers, enablers, implementation strategies, and evaluation methods for implementing PROMs/PREMs across a range of patient populations and care settings. Below we describe lessons learned, recommendations, and areas in need of future research.

### Relevance of IS approaches for PROMs/PREMs implementation

Our cross-study analysis demonstrates that IS approaches are largely harmonious with PROMs/PREMs implementation, although no single framework or theory fully captures their nuances. Multilevel frameworks and theories are necessary for PROM/PREM implementation given its complexity. IS theoretical approaches are not prescriptive but can be used flexibly, potentially in combinations, to suit specific contexts; multiple frameworks were used in two case studies presented here to emphasize different domains.

CFIR was the most commonly used framework, applied in three case studies during pre-implementation and implementation stages. CFIR is fairly comprehensive for categorizing barriers and enablers, but it does not specify mechanisms by which strategies might improve implementation effectiveness. Given the broad nature of CFIR, the pain clinic case study [30] found CFIR captured more barriers than TDF for clinician knowledge and perceptions. The case study by van Oers et al. [32] noted difficulty in operationalizing concepts in CFIR because of overlapping subdomains and difficulty in classifying PROM/PREM characteristics (e.g., item content, psychometric properties) in the subdomain “characteristics of the innovation,” suggesting modifications to CFIR or additional frameworks may be needed to capture PROM/PREM nuances. The availability of CFIR’s tool for matching implementation strategies to barriers also contributed to its perceived utility, although the usefulness of the matching tool in practice was unclear.

Of the widely used IS frameworks and theories described in this paper, Normalization Process Theory (NPT) [45, 46] is distinct in proposing mechanisms for sustained uptake of innovations. NPT’s core constructs of coherence/sense-making, cognitive participation, collective action, and reflexive monitoring overlap with domains from other IS frameworks and implementation strategies used in case studies (see Table 5). The applicability of these general constructs to specific PROMs/PREMs implementation efforts should be tested in future studies. If they prove to be robust in new settings, these potential drivers of implementation could inform a more universal strategy for increasing the uptake of PROMs/PREMs in routine care.

**Table 5:** General strategies for implementing PROMs/PREMs in routine care (derived from Normalization Process Theory [NPT] [45, 46])

Core constructs from NPT [45, 46], adapted for PROMs/PREMs implementation	1. Coherence: Assess understanding of PROMs/PREMs in context What are PROMs/PREMs, and why should clinical teams use them?	2. Cognitive participation: Engage stakeholders in communities of practice Who will do what for routine use of PROMs/PREMs in clinical care?	3. Collective action: Identify barriers and facilitators What helps or hinders the use of PROMs/PREMs in clinical care? Whom do these factors affect?	4. Reflexive monitoring: Evaluate understanding of routine PROMs/PREMs use What did we learn about using PROMs/PREMs in clinic? Will we keep doing it?
Overlap with relevant domains from widely used implementation science frameworks	KTA: Identify problem, Select and Review Knowledge i-PARIHS: Innovation (how it is perceived by various stakeholders), Recipient CFIR: Intervention characteristics (e.g., evidence strength and quality, relative advantage, adaptability, complexity), Characteristcs of individuals (e.g., knowledge and beliefs about the intervention) TDF: Knowledge, beliefs and capabilities, social/professional role and identity, beliefs about consequences RE-AIM: Effectiveness (longer-term impacts e.g., quality of life) Proctor’s outcomes: Appropriateness, Cost, Feasibility (stakeholder perceptions)	KTA: Adapt knowledge to local context (involve local stakeholders) i-PARIHS: Recipient (identify key stakeholders including patients), Facilitation (regular meetings with clinic) CFIR: Leadership engagement (under Inner setting), Process (e.g., engaging, opinion leaders, internal implementation leaders, champions, external change agents) TDF: Skills, memory, attention and decision, emotion, behavioral regulation, intentions, goals, optimism RE-AIM: Reach, Adoption (numbers of patients and champions willing to participate in implementation) Proctor’s outcomes: Acceptability, Adoption, Penetration	KTA: Assess barriers to knowledge use i-PARIHS: Innovation (how it is adapted to work in local contexts), Context (inner setting and outer setting) CFIR: Outer setting (e.g., patient needs and resources, external policies and incentives), Inner setting (e.g., networks and communication, culture, relative priority, organizational incentives, available resources, access to knowledge and information) TDF: Reinforcement, environmental context and resources, social influences RE-AIM: Maintenance (normalized 6 months after introduction) Proctor’s outcomes: Feasibility, Cost	KTA: Monitor knowledge use, Evaluate outcomes i-PARIHS: Facilitation, Organizational Readiness to Change assessment CFIR: Reflecting and evaluating (under Process) RE-AIM: Implementation (fidelity) Proctor’s outcomes: Sustainability
Implementation strategies identified in case studies	• Stakeholder engagement • Provide evidence about clinical validity of PROMs/PREMs	• Training workshops • Workflow redesign • Implementation support team	• Context assessments • Technology support • Practice facilitator	• Annual evaluation meetings with clinics • Audit and feedback

*PROM* electronic patient-reported outcome measure, *PREM* electronic patient-reported experience measure, *CFIR* consolidated framework for implementation research, *i-PARIHS* integrated framework for promoting action on research implementation in health services, *KTA* knowledge to action, *TDF* theoretical domains framework, *NPT* normalization process theory, *RE-AIM Reach* effectiveness, adoption, implementation, maintenance framework

We recommend choosing an IS framework or theory based on fit for purpose [24, 25]. Research focused on identifying and categorizing barriers and enablers may benefit from descriptive frameworks like i-PARIHS or CFIR, which also provide lists of evidence-based implementation strategies. Research describing translation processes could use a process model like KTA or implementation theory like NPT. All case studies combined descriptive frameworks or process models/theory with evaluation frameworks. Existing implementation toolkits can aid in decisions on which frameworks, theories, and implementation strategies might be appropriate for specific PROM/PREM projects [55, 56].

### Consistent barriers, context-specific enablers, and tailored implementation strategies

A key finding from our cross-study analysis was that barriers were consistent across populations and care settings, but enablers were context specific. Barriers included technology limitations, uncertainty about ease and benefit of using PROMs/PREMs, concerns about potential negative impacts, and competing demands within established clinical workflows. These barriers are also consistent with existing literature [18–21], including ISOQOL’s PROM User Guides [20], suggesting that clinics and implementation teams should include these known barriers in their pre-implementation planning process.

While barriers in case studies were consistent, an important finding from our analysis was that enablers were context specific and based on local clinic resources. The observed variation in PROM/PREM enablers indicates the potential for tailored solutions for clinics. A common enabling factor was an existing PROM/PREM technology system with automated features. More unique enablers capitalized on local resources, such as providing clinics with implementation funding, media campaigns, and having physician champions teach part of PROM/PREM training sessions. Future research should examine whether co-designing PROMs/PREMs implementation strategies with clinics improves implementation effectiveness and patient outcomes.

The variation we observed in implementation strategies may have more to do with implementation stage and local clinic needs than particular care settings or populations. For example, all case studies found that engaging clinicians during development of implementation strategies was critical; but the PREM case study [33] also found it useful to engage quality improvement specialists because that was an available resource. Manalili et al. [33] noted that clinics may need support in building capacity for quality improvement before PROMs/PREMs can be introduced. Ahmed et al. [30] used a standardized process called intervention mapping [57, 58] to map barriers to evidence-based strategies.

Common PROM/PREM implementation strategies in case studies matched most of Powell and colleagues’ “Expert Recommendations for Implementing Change” (ERIC) [27], suggesting there are critical implementation strategies that need to be conducted regardless of setting or population (e.g., training clinic teams and providing implementation support). For example, Skovlund et al. [59] developed a PROM training tool for clinicians that may be useful as an implementation strategy. Future research should determine key implementation strategies that enable higher uptake of PROMs/PREMs in clinics.

Consistent implementation strategies across case studies included providing technology and workflow support to clinics, but it ranged from low to high intensity. Roberts et al. [31] found that having a dedicated implementation support role (nurse-trained scientist) was critical for maintaining momentum during pre-implementation and implementation phases in cancer clinics. Across case studies, clinics needed flexibility and support in adapting their workflow, but there is no corollary listed in the ERIC strategies. We agree with Perry et al. [60] who recommended adding workflow assessment and care redesign to the ERIC [27] list based on their empirical data. Future research is needed on the optimal level and intensity of implementation support for successful PROM/PREM implementation.

Case studies were inconsistent in the level of detail provided about implementation strategies, which may inhibit replication. We recommend PROM/PREM IS studies follow the “Standards for Reporting Implementation Studies” (StaRI) guidelines [61]. A framework called “Action, actor, context, target, time (AACTT)” [62] may be useful as a reporting tool for describing PROM/PREM implementation strategies. Leeman et al. [28] also recommend defining implementation strategies a priori and specifying who will enact the strategy (actor) and the level and determinants that will be targeted (action targets).

### Need for consistent and robust measurement in IS evaluation

In the case studies, we highlighted inconsistencies in IS evaluation. We therefore developed IS metrics specific to PROM/PREM implementation to support reporting and standardization (Table 4). These metrics are not questionnaires, but rather percentages of how many clinics achieve milestones like completing implementation activities. Our metrics advance the field of IS by being one of the first to describe separate metrics for evaluating perceptions of a health innovation vs. implementation effectiveness. Future research is needed to build validity and reliability evidence for these metrics.

A related issue is that many IS questionnaires assessing Proctor’s constructs and contextual factors lack psychometric testing, validity and reliability evidence, and short forms. Systematic reviews of IS questionnaires [53, 54, 63–65] show that information on reliability is unavailable for half of IS instruments and > 80% lacked validity evidence [54]. IS questionnaires tend to be long (30+ items), so their utility in busy clinics may be limited [66]. They also have unclear relevance for PROMs/PREMs implementation. With notable exceptions [65, 67, 68], few IS scales have published psychometric properties [54]. For example, one exception with published classical test theory data developed short forms to assess acceptability, appropriateness, and perceived feasibility across implementation initiatives [68]. These generic short forms were used in the pain clinic case study [30], and they are being tested in cancer care PROM implementation in the U.S. National Cancer Institute’s IMPACT consortium [69].

It is unknown how many IS questionnaires meet psychometric standards and whether new instruments need to be developed for PROMs/PREMs implementation and thus, scale reviews specific to PROMs/PREM implementation are needed. Funding agencies interested in PROM/PREM implementation should consider requesting proposals to generate this critical psychometric evidence to ensure standardization and replicability. Ideally, if shorter versions of existing IS questionnaires could be developed, comparisons with health care IS studies outside of PROMs/PREMs may be possible.

### Why implementation theory is needed

Increasing the use of IS in PROM/PREM implementation studies will help advance our collective understanding of how, why, and in what circumstances IS frameworks and implementation strategies produce successful implementation (or not). Mechanisms of change may differ between active implementation and sustainability, and even between PROMs and PREMs. Future research should explicitly test hypothesized pathways through which implementation strategies exert their effects on implementation outcomes, care delivery, and patient outcomes. Figure 1 shows that mediators (or potentially moderators) in these pathways are contextual factors. Future research is needed to determine which contextual factors matter for PROM/PREM implementation and how best to assess them.

Pathways linking strategies with IS outcomes and clinical outcomes can be tested with stepped wedge designs, pragmatic trials, and theory-driven mixed methods such as realist evaluation [6, 70–72]. Realist evaluation seeks to understand how context shapes the mechanisms through which a health care innovation works. Realist evaluation recognizes that complex interventions (such as those informed by IS) are rarely universally successful, because clinic context plays a significant role in shaping their uptake and impact. This is consistent with our finding that PROM/PREM clinic enablers had more variation than barriers in case studies. While RCTs and pragmatic trials are useful to evaluate the net or average effect of an intervention, realist evaluation could help clarify why specific implementation strategies work in some contextual conditions but not others [6, 70–72], and could complement other IS approaches. Research on the “how” and “why” of implementation processes will help move the field beyond simply identifying barriers and enablers of PROMs/PREMs implementation, to proactively designing and comparing implementation strategies.

## Conclusion

In four case studies, IS frameworks were used to systematize barriers to PROM/PREM implementation, to develop implementation support strategies for clinic teams, and to evaluate implementation effectiveness. Barriers to PROM/PREM implementation were remarkably consistent across patient populations and care settings, suggesting that implementation strategies addressing contextual factors may have wide-reaching impact on implementation effectiveness. Flexibility in promoting clinic-specific enablers was also highlighted, as was a need for consistency in evaluating PROM/PREM implementation effectiveness. Theoretically guided studies are needed to clarify how, why, and in what circumstances IS approaches lead to successful PROM/PREM integration and sustainability.

## Electronic supplementary material

Below is the link to the electronic supplementary material.

Supplementary file1 (DOCX 13 kb)

## Footnotes

1 We use the term “facilitators” in this paper series to designate the implementation support person working with clinics, rather than the classic use of the word in IS to mean enablers, such as clinic resources.
