# Supplementary material for: Using an implementation science approach to implement and evaluate patient-reported outcome measures (PROM) initiatives in routine care settings
Source: Qual Life Res. 2020 Jul 10;30(11):3015–33. doi: 10.1007/s11136-020-02564-9 (PMC8528754; doi:10.1007/s11136-020-02564-9)
Supplement: Supplementary file 1 — Supplementary file1 (DOCX 13 kb) [file 11136_2020_2564_MOESM1_ESM.docx]

**Online Resource 1**

**ISOQOL PROMs/PREMs in Clinical Practice Implementation Science Work Group
(in alphabetical order)**

| **Name** | **University or Organization** | **Country** |
| --- | --- | --- |
| Sara Ahmed, PhD | McGill University | Canada |
| Joanne Greenhalgh, PhD | University of Leeds | U.K. |
| Elizabeth Gibbons, MSc | Clinical Outcome Solutions | U.K. |
| Lotte Haverman, PhD | Amsterdam UMC | Netherlands |
| Kimberly Manalili, MPH, PhD (c) | University of Calgary | Canada |
| Caroline Potter, PhD | Oxford University | U.K. |
| Natasha Roberts, PhD (c) | Queensland University of Technology | Australia |
| Maria Santana, PhD | University of Calgary | Canada |
| Angela M. Stover, PhD | University of North Carolina at Chapel Hill | U.S. |
| Hedy van Oers, PhD | Amsterdam UMC | Netherlands |

**Article title**: Using an Implementation Science Approach to Implement and Evaluate Patient Reported Outcome Measures (PROM) Initiatives in Routine Care Settings

**Journal name**: Quality of Life Research

**Article author names and affiliation(s)**:

Angela M. Stover, PhD ^1,2^ *

Lotte Haverman, PhD ^3^

Hedy A. van Oers, PhD ^3^

Joanne Greenhalgh, PhD ^4^

Caroline M. Potter, DPhil ^5^

On behalf of the ISOQOL PROMs/PREMs in Clinical Practice Implementation Science Work Group ^6^

1. Department of Health Policy and Management, University of North Carolina at Chapel Hill, USA

2. Lineberger Comprehensive Cancer Center, University of North Carolina at Chapel Hill, USA

3. Psychosocial Department, Emma Children’s Hospital, Amsterdam UMC, University of Amsterdam, NL

4. School of Sociology and Social Policy, University of Leeds, UK

5. Nuffield Department of Population Health, University of Oxford, UK

6. See Online Resource 1

***E-mail address of corresponding author**: [stoveram@email.unc.edu](mailto:stoveram@email.unc.edu)
